# Acetyl-CoA Carboxylase Inhibitors for Nonalcoholic Fatty Liver Disease: A Systematic Review and Meta-Analysis of Randomized Controlled Trials

**DOI:** 10.3390/ph18091276

**Published:** 2025-08-27

**Authors:** Nurina Hasanatuludhhiyah, Arifa Mustika, Viskasari P. Kalanjati, Muhammad Miftahussurur, Naoto Uemura

**Affiliations:** 1 Doctoral Program of Medical Science, Faculty of Medicine, Universitas Airlangga, Surabaya 60131, Indonesia; nurina.hasanatuludhhiyah-2021@fk.unair.ac.id; 2 Department of Anatomy, Histology, and Pharmacology, Faculty of Medicine, Universitas Airlangga, Surabaya 60131, Indonesia; viskasari-p-k@fk.unair.ac.id; 3 Department of Clinical Pharmacology and Therapeutics, Faculty of Medicine, Oita University, Yufu 879-5593, Oita, Japan; uemura@oita-u.ac.jp; 4 Division of Gastroentero-Hepatology, Department of Internal Medicine, Faculty of Medicine—Dr. Soetomo Teaching Hospital, Universitas Airlangga, Surabaya 60286, Indonesia; muhammad-m@fk.unair.ac.id; 5 Helicobacter pylori and Microbiota Study Group, Institute of Tropical Disease, Universitas Airlangga, Surabaya 60115, Indonesia

**Keywords:** adverse events, fibrosis, firsocostat, hepatitis, hypertriglyceridemia, liver fat, NAFLD, MASLD, metabolic dysfunction

## Abstract

**Background/Objectives**: Acetyl-CoA carboxylase (ACC) inhibitors block the initial step of de novo lipogenesis and potentially ameliorate liver pathology in nonalcoholic fatty liver disease (NAFLD). However, increased expression of glycerol-3-phosphate acyltransferase 1 resulting from reduced PUFA may cause hypertriglyceridemia. This systematic review and meta-analysis assessed the efficacy and safety of dual ACC 1/2 inhibitors in adult NAFLD patients, either with or without metabolic dysfunction. **Methods**: Six databases were searched for randomized controlled trials (RCTs). The primary outcomes were changes in liver fat and fibrosis. Study quality was assessed using the RoB 2 tool. Pooled mean differences (MDs) and odds ratios (ORs) with 95% confidence intervals (CIs) were calculated using a fixed-effects model. **Results**: Six RCTs comprising 655 participants were included; most had low risk of bias. Interventions included firsocostat, clesacostat, and combined regimens with semaglutide, selonsertib, and cilofexor or ervogastat. Compared with placeo, ACC inhibitor monotherapy significantly reduced liver fat (mean difference [MD]: −48.38; 95% CI: −58.54 to −38.22; *p* < 0.00001) and ALT (MD: −16.07; 95% CI: −24.97 to −7.17; *p* = 0.0004) but increased ALP (MD: 11.95; 95% CI: 6.98, to 16.92; *p* < 0.00001) and GGT levels (MD: 23.90; 95% CI: 12.58 to 35.23; *p* < 0.0001). Hypertriglyceridemia risk was markedly elevated (odds ratio [OR]: 10.33; 95% CI: 4.93 to 21.65; *p* < 0.00001). No significant improvement in fibrosis was observed by magnetic resonance elastography. Serious adverse events were infrequent, and overall treatment-emergent adverse events were comparable between groups; however, the incidence of hypertriglyceridemia was consistently more frequent with ACC inhibitors. **Conclusions**: Dual ACC 1/2 inhibitors reduce hepatic steatosis and ALT levels but do not improve fibrosis. Their consistent association with hypertriglyceridemia raises concerns regarding potential long-term cardiometabolic risks, particularly in NAFLD patients with metabolic dysfunction.

## 1. Introduction

Nonalcoholic fatty liver disease (NAFLD) has emerged as a global epidemic, driven by rising rates of obesity and type 2 diabetes, the two primary risk factors [1]. Currently, NAFLD is the most common liver disease worldwide and a leading cause of cirrhosis and hepatocellular carcinoma-related mortality. Despite significant advances in understanding the pathophysiology of NAFLD, pharmacological treatments remain limited [2,3]. Recently redefined as metabolic dysfunction-associated steatotic liver disease (MASLD), NAFLD represents a spectrum of liver disease, ranging from simple steatosis to steatohepatitis, fibrosis, and, in severe cases, cirrhosis, which can progress to carcinoma or liver failure. Although isolated steatosis is often considered benign, ongoing cardiometabolic risk factors can accelerate liver damage [4]. In addition to liver-related complications, cardiovascular events largely contribute to mortality in patients with NAFLD [5]. The transition from NAFLD to MASLD mandates focusing on cardiometabolic risk for the diagnosis rather than the exclusion of other causes.

The pathogenesis of NAFLD is best described by the recent multi-hit model, which implicates a complex interplay of genetic and environmental factors, driven by dysregulated lipid and glucose metabolism, meta-inflammation, gut dysbiosis, and oxidative stress [6]. Lipotoxicity represents one of the fundamental mechanistic axes in this multi-hit model, linking the hallmark feature of lipid accumulation to mitochondrial dysfunction and oxidative stress [7]. The intrahepatic build-up of toxic lipids results from an imbalance between influx and anabolism, with catabolism and secretion of lipid species. De novo lipogenesis (DNL) greatly contributes to hepatocyte triglyceride accumulation in NAFLD, with overabundant fatty acid production from acetyl-CoA [8]. Once TG accumulation has exceeded buffer capacity in hepatocytes, toxic lipid metabolites elicit cellular injury and inflammatory response, activating the profibrogenic pathway [9]. Acetyl-CoA carboxylase (ACC) catalyzes the initial committed step of the DNL pathway [10]. The two isoforms of acetyl-CoA carboxylase serve a catalytic function, converting acetyl-CoA into malonyl-CoA. ACC1, located in the cytosol, is accountable for producing the substrate of intrahepatic fatty acid synthesis, while ACC2, situated in the outer mitochondrial membrane, produces malonyl CoA, which allosterically inhibits carnitine palmitoyltransferase 1 (CPT-1). Consequently, fatty acid entry to mitochondria is reduced, thereby modulating β-oxidation of fatty acids. These suggest a key regulatory role of ACC in fatty acid anabolism and catabolism [10,11].

Preclinical studies have shown that dual ACC 1/2 inhibitors effectively reduced liver inflammation, reversed fibrosis, and improved liver function in NASH models [12]. Furthermore, ND-654, a liver-directed ACC 1/2 allosteric inhibitor, effectively lowered hepatocellular carcinoma in rodent models, supplementing evidence that this agent is a promising therapeutic for decelerating NAFLD progression [13]. ACC inhibitors have also been shown to improve hepatic insulin resistance. However, unexpected hypertriglyceridemia occurred after long-term treatment, raising concerns for deleterious effects on cardiometabolic factors that can lead to cardiovascular events [14]. Several randomized clinical trials (RCTs) have also shown promising therapeutic advantages of these agents, yet the findings regarding their efficacy are inconsistent, and safety concerns have emerged [15]. Given these uncertainties, a systematic review and meta-analysis are necessary to consolidate the available data on the efficacy and safety of ACC inhibitors in NAFLD patients. To our knowledge, no comprehensive meta-analysis has thoroughly assessed the efficacy of ACC 1/2 dual inhibitors in reducing liver fat and fibrosis or examined their safety profile in this context. Therefore, we conducted a systematic review and meta-analysis of RCTs to evaluate the efficacy and safety of ACC 1/2 dual inhibitors in patients with NAFLD who either have metabolic dysfunction or do not.

## 2. Materials and Methods

### 2.1. Study Design and Protocol Registration

A systematic review and meta-analysis were undertaken to evaluate the efficacy and safety of ACC inhibitors in adult patients with NAFLD. This study followed the guideline of preferred reporting items for systematic reviews and meta-analysis protocol (PRISMA) [16],and was prospectively registered on the PROSPERO database, ID: CRD42024541418 (https://www.crd.york.ac.uk/PROSPERO/view/CRD42024541418 (accessed on 5 August 2025)).

### 2.2. Eligibility Criteria

Randomized controlled trials evaluating the efficacy and safety of ACC 1/2 dual inhibitors in patients with NAFLD (who either have metabolic dysfunction or do not) were included. Review articles, case reports, case series, letters to the editor, and conference abstracts were not included in this review. We formulated the clinical questions for this systematic review based on the PICO framework, as shown in Supplementary Table S1. The primary outcomes included changes in liver fat content and fibrosis measured by magnetic resonance imaging, proton density fat fraction (MRI-PDFF), and magnetic resonance elastography (MRE), respectively. The secondary outcomes included other measures of liver fibrosis, changes in biochemistry parameters (AST, ALT, ALP, GGT, total bilirubin, glucose, HbA1c, insulin, etc.), and reported adverse events.

### 2.3. Search Strategy

A systematic search was conducted across multiple databases, including PubMed, Scopus, Web of Science, ProQuest, the Cumulative Index to Nursing and Allied Health Literature (CINAHL) via EBSCO, and the Cochrane Central Register of Controlled Trials (CENTRAL). The search encompassed all studies published up to July 2025. The search strategy utilized a combination of Medical Subject Headings (MeSH) and free-text terms to retrieve relevant studies comprehensively. These terms were structured around the PICO framework, and the keywords were adapted to the specific indexing systems of each database, as presented in the Supplementary Table S2.

### 2.4. Study Selection and Screening

The search results from all databases were organized using Google Sheets (Google LLC, Mountain View, CA, USA). The deduplication process was done manually by two investigators (NH and AM). NH and AM independently screened the titles and abstracts of all identified studies for eligibility. Full-text articles of potentially eligible studies were retrieved and assessed based on predefined inclusion and exclusion criteria. Inter-rater reliability was assessed using Cohen’s kappa coefficient. Any discrepancies between the reviewers were resolved through discussion with VPK. The study selection process was illustrated using a PRISMA 2020 flowchart, which included the reasons for exclusion at each stage [16].

### 2.5. Data Extraction

NH and AM extracted data using a pre-defined data extraction form. The extracted information included (1) author and publication year; (2) study location; (3) participant characteristics, such as sample size, age, percentage of female participants, weight, BMI, proportion of DM, fibrosis stage, and baseline liver biochemistry and triglyceride levels; (4) details of the intervention, including regimen, dosage, and duration; and (5) outcome measures, such as means and standard deviations (SD). For studies reporting outcomes in formats that could not be directly extracted (e.g., confidence intervals), the data were transformed into the desired format using methods provided by Cochrane’s Handbook [17].

### 2.6. Risk of Bias Assessment

The quality assessment of included studies was evaluated using the Cochrane Risk of Bias 2 (RoB 2) tool (https://methods.cochrane.org/bias/resources/rob-2-revised-cochrane-risk-bias-tool-randomized-trials (accessed on 5 August 2025)), which assesses five domains: randomization process, deviations from intended interventions, missing outcome data, measurement of the outcome, and selection of the reported result. Each domain was rated as “low risk”, “some concerns”, or “high risk” of bias [18]. Two independent reviewers conducted the assessments, and disagreements were resolved by consensus involving the third reviewer.

### 2.7. Study Variables

The independent variable in this study was ACC 1/2 dual inhibitors, i.e., firsocostat (GS-0976) and clesacostat (PF-0522134). Efficacy and safety outcomes upon administration of these agents in adult NAFLD patients were evaluated through clinical, radiological, and laboratory measures. The effects of the intervention on hallmark liver pathologies of steatosis and fibrosis were primarily assessed by MRI-PDFF and MRE, respectively. MRI-PDFF measurement on liver fat content reliably assesses the levels of steatosis in NAFLD patients [19]. MRE is an MRI-based technique that quantitatively images the increased stiffness of hepatic parenchyma, which is a direct result of liver fibrosis [20]. Additional measures for fibrosis were also included in this review. They comprise radiological assessments such as VCTE and blood-based biomarkers or scores, i.e., TIMP-1, ELF score, and fibrotest score. The laboratory parameters include liver function tests that can reflect the presence of hepatic injury, namely serum levels of aspartate aminotransferase (AST), alanine aminotransferase (ALT), alkaline phosphatase (ALP), gamma-glutamyl transferase (GGT), and bilirubin. We also evaluated the metabolic parameters, including blood glucose, HbA1c, insulin, and lipid panel. Safety outcomes were reported as treatment-emergent adverse events and treatment-emergent laboratory abnormalities.

### 2.8. Statistical Analysis

We used Hedges’ g method to estimate the pooled mean difference (MD) with 95% confidence intervals (CIs) between intervention and control groups. Meta-analyses were conducted using a fixed-effects model. Heterogeneity was evaluated using Cochran’s Q test and the I^2^ statistic, with I^2^ values of 25%, 50%, and 75% indicating low, moderate, and high heterogeneity, respectively. Sensitivity analyses were conducted using the leave-one-out method to assess the robustness of the pooled estimates. All statistical analyses were performed using Review Manager (RevMan) version 5.4 software. *p*-values of < 0.05 were considered statistically significant.

## 3. Results

### 3.1. Overview of Study Selection

**Figure 1:**
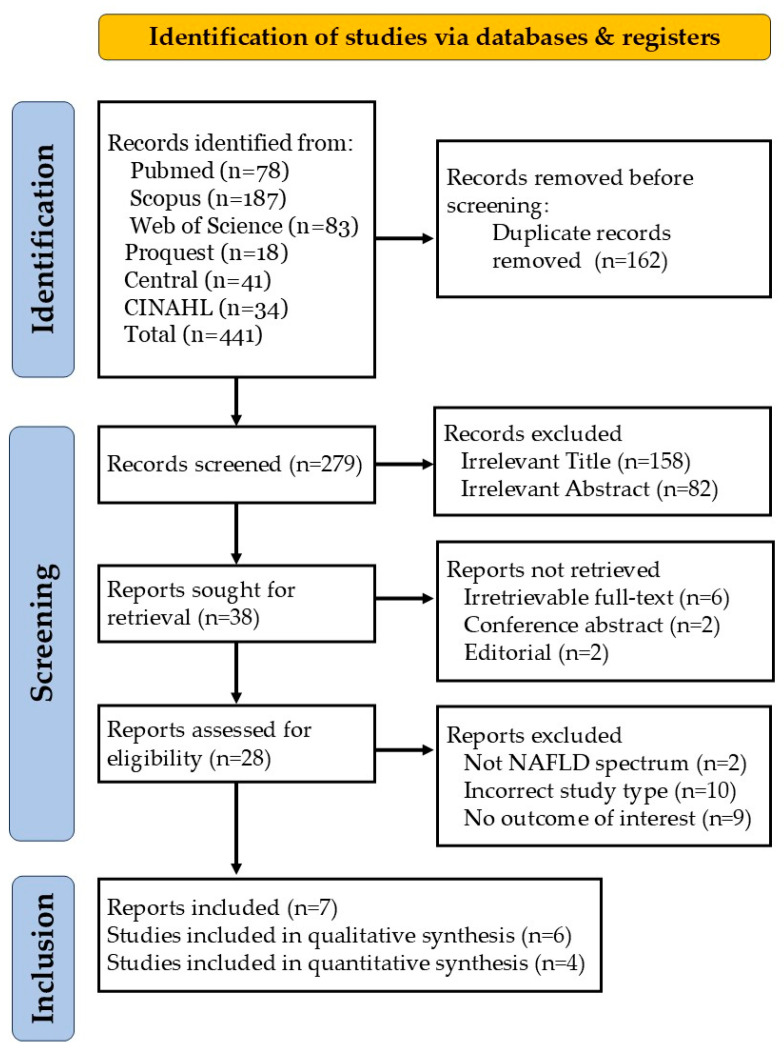
PRISMA flowchart of the study selection process.

A PRISMA flowchart illustrating the study selection process is shown in Figure 1. The initial search across six databases yielded 441 records of publications dated from March 2008 to July 2025. After manually identifying and removing 162 duplicate records, 279 unique records remained. Based on their titles and abstracts, 158 records with irrelevant titles—for example, animal studies—were excluded, followed by 82 exclusions after the abstract review. The remaining 38 records were sought for the full-text reports. Ten articles, including those with unavailable full-texts, conference abstracts, and editorials, could not be retrieved. The full texts of the remaining 28 reports were thoroughly reviewed, and 21 were further excluded for the following reasons: not part of the NAFLD spectrum (*n* = 2), incorrect study design (*n* = 10), or lack of relevant outcomes (*n* = 9). This screening process ultimately resulted in the inclusion of six studies for qualitative analysis and four studies for quantitative analysis. The Cohen’s kappa for title, abstract, and full-text screening was 0.87, 0.83, and 0.91, respectively, indicating a high agreement between reviewers.

### 3.2. Characteristics and Outcomes of Included Studies

The characteristics of the included studies are summarized in Table 1. These studies, which were in phase 2, 2a, and 2b clinical trials, involved 655 adults, with a mean age ranging from 52.5 to 61.4 years. The proportion of female participants varied from 46.5% to 69.76%. All studies were conducted in the USA, and two were conducted in multiple countries of the Western and Asian regions. All studies used the NASH Clinical Research Network (CRN) classification to define NAFLD. All but one study set up a criterion of fibrosis stage for inclusion, with two of these studies enrolling subjects with cirrhosis or at stage F4. Three RCTs added the presence of ≥2 of 5 signs of metabolic dysfunction into the eligibility criteria, making the population characteristics fit the requirements of MASLD diagnosis. The percentage of subjects with type 2 DM was high, reaching 72% of the studied population in one RCT by Loomba et al. (2021) [21]. Moreover, the subjects were mostly obese, with the average BMIs ranging from 31.9 ± 5.5 to 36.6 ± 4.7. Four out of six studies evaluated ACC inhibitors in combination with other agents, either semaglutide, a glucagon-like peptide-1 (GLP-1) agonist, Ervogastat, a diacylglycerol acyltransferase (DGAT2) inhibitor, Cilofexor, a nonsteroidal farnesoid X receptor (FXR) agonist, or selonsertib, an apoptosis signal-regulating kinase-1 (ASK1) inhibitor.

The outcomes of the six studies are summarized in Table 2. Four studies reported glucose-related outcomes, including fasting blood glucose, fasting insulin, HOMA-IR, and HbA1c. Four studies reported changes in liver enzymes. All studies reporting MRI-PDFF change relative to baseline values documented significant reductions in liver fat content in groups treated with ACC inhibitors, either alone or as a combined regimen. A study evaluating clesacostat monotherapy by Calle et al. (2021) suggested a dose-dependent reduction of liver fat. Administration of clesacostat 25 mg for 16 weeks produced a higher percentage of MRI-PDFF relative reduction and a higher proportion of subjects with clinically significant improvement of steatosis compared with clesacostat 10 mg [22]. None reported a significant reduction in liver stiffness as measured by MRE. Nonetheless, two studies reported significant improvement in other fibrosis markers. A study by Loomba et al. (2021) documented significant changes in enhanced liver fibrosis (ELF) scores (LSM (95% CI) −0.1 (−0.4, 0.1) vs. 0.3 (0.1, 0.6); *p* = 0.010) and liver stiffness measured by VCTE (LSM (95% CI) −6.3 (−9.6, −3.0) vs. −1.2 (−4.1, 1.8) kPa; *p* = 0.021) [21]. A significant reduction in liver stiffness measured by transient elastography (TE), tissue inhibitor metalloproteinase (TIMP-1), and Procollagen III-N-terminal peptide (P-III-NP) levels was reported in a study by Loomba et al. (2018) [23].

In general, administration of ACC inhibitors did not produce significant effects on glucose metabolism. A study by AlKhouri et al. (2022) found no significant differences in fasting glucose and HbA1c between the group receiving a combination of semaglutide + firsocostat and the group receiving semaglutide alone [24]. Firsocostat alone showed no significant effects compared to placebo on fasting insulin, glucose, and HbA1c [21,23]. However, a combination of Cilofexor and firsocostat improved glycemic parameters, as indicated by significantly reduced fasting insulin levels relative to placebo. Moreover, this combination regimen tended to show better reductions in fasting insulin and HOMA-IR, with lower increases in fasting glucose and HbA1c than Cilofexor alone [21]. Interestingly, an improvement in HbA1c was shown after 16 weeks of clesacostat monotherapy. This effect tended to be dose-dependent and was more pronounced in a subgroup of subjects with T2DM [22].

**Table 1:** Characteristics of the included studies.

Trial Phase Author (Year)	Study Location	Characteristics of Population	Regimen, Dose		Sample Size				Age (Year)	Female (%)	Weight (Kg)/ BMI	T2DM (%)	Fibrosis	AST/ALT (U/L)	ALP/GGT (U/L)	TG (mg/dL)
			Intervention	Control	Intervention		Control									
					Pre	Post	Pre	Post								
Phase 2 Al Khouri et al. (2022) [24]	USA	Adults with NAFLD	Semaglutide 0.24–24 mg SC (escalated dose over 16 weeks) once weekly + Firsocostat 20 mg oral, once daily	Semaglutide 0.24–24 mg SC (escalated dose over 16 weeks), once weekly	22	20	21	18	53.5 ± 11.5	69.76	97 ± 25.1/ 33.7 ± 5.3	55.8	F2–F3 or TE ≥ 7 kPA	AST: 43 (26–51) vs 50 (36–61) ALT: 45 (29–76) vs. 60 (48–98)	ALP: 82 (64–105) vs. 78 (62–92) GGT: 38 (26–94) vs. 35 (30–56)	160 (116–212) vs. 167 (104–230)
Phase 2a Calle et al. (2021 a) [22]	Australia Canada Israel Poland Taiwan USA	Adults with NAFLD and metabolic syndrome ^a^	Clesacostat 10 mg, oral, once daily	Placebo	62	55	61	54	53.3 ± 11.7	57.5	N/A/ 33.9 ± 5.3	40.9	N/A	AST: 39.9 ± 23.1 vs 42.2 ± 27.6 ALT: 59.0 ± 30.6 vs. 58.9 ± 48.6	ALP: 82.0 ± 25.5 vs. 76.8 ± 25.3 GGT: 58.2 ± 36.6 vs. 56.4 ± 42.5	172.2 ± 83.3 vs. 178.2 ± 84.9
			Clesacostat 25 mg, oral, once daily		58	48							N/A	AST: 41.9 ± 18.9 vs 42.2 ± 27.6 ALT: 57.8 ± 29.8 vs. 58.9 ± 48.6	ALP: 77.7 ± 22.9 vs. 76.8 ± 25.3 GGT: 50.0 ± 35.6 vs. 56.4 ± 42.5	165.4 ± 78.7 vs. 178.2 ± 84.9
Phase 2a Calle et al. (2021 b) [22]	USA	Adults with NAFLD and metabolic syndrome ^a^	Clesacostat 15mg, oral, twice daily	Placebo	29	22	14	13	54.6 ± 11.0	44.6	N/A/ 35.7 ± 5.1	N/A	N/A	AST: 24.0 ± 8.1 vs 25.9 ± 9.5 ALT: 31.9 ± 15.7 vs. 35.0 ± 23.0	ALP: 85.8 ± 32.3 vs. 91.9 ± 37.4 GGT: 32.9 ± 17.0 vs. 38.0 ± 25.0	214.2 ± 134.9 vs. 164.2 ± 82.1
			Clesacostat 15mg + Ervogastat 300mg, oral, twice daily		28	26						N/A	N/A	AST: 23.5 ± 7.4 vs 25.9 ± 9.5 ALT: 32.8 ± 16.1 vs. 35.0 ± 23.0	ALP: 84.0 ± 18.3 vs. 91.9 ± 37.4 GGT: 34.6 ± 19.3 vs. 38.0 ± 25.0	175.3 ± 66.8 vs. 164.2 ± 82.1
			Clesacostat 15mg + Ervogastat 300mg, oral, twice daily	Ervogastat 300mg oral, twice daily	28	26	28	24	53.5 ± 10.4	46.4	N/A/ 36.6 ± 4.7	N/A	N/A	AST: 23.5 ± 7.4 vs 26.0 ± 8.5 ALT: 32.8 ± 16.1 vs. 36.0 ± 18.5	ALP: 84.0 ± 18.3 vs. 82.4 ± 15.8 GGT: 34.6 ± 19.3 vs. 37.2 ± 17.7	175.3 ± 66.8 vs. 173.3 ± 90.5
Phase 2a Dandan et al. (2023) Lawitz et al. (2023) [25,26]	USA	Adults with NAFLD and metabolic syndrome^a^	Firsocostat 20 mg, oral, once daily	Selonsertib 18 mg, oral, once daily	10	10	10	10	54.7 ± 9.7	76.7	N/A	N/A	≥F2 VCTE ≥ 9.9 kPa or MRE ≥ 2.88 kPa	N/A	N/A	N/A
			Firsocostat 20 mg, oral, once daily	Cilofexor 30 mg, oral, once daily	10	10	10	10								
			Selonsertib 18 mg + Firsocostat 20 mg, oral, once daily	Selonsertib 18 mg, oral, once daily	20	20	10	10	52.5 ± 11.0	68.3	N/A	N/A		N/A	N/A	N/A
			Cilofexor 30 mg + Firsocostat 20 mg, oral, once daily	Cilofexor 30 mg, oral, once daily	20	19	10	10								
Phase 2b Loomba et al. (2021) [21]	USA Canada Australia New Zealand Honkong	Adults with NAFLD	Firsocostat 20 mg, oral, once daily	Placebo	40	33	39	38	61.4 ± 8.5	65.8	93.4 ± 22.4/34.5 ± 6.7	72.2	F3–F4 or VCTE ≥ 14 kPA and ELF ≥ 9.8	AST:41 (33, 59) vs. 41 (27, 62) ALT: 47 (32, 62) vs. 44 (29, 61)	ALP: 73 (64, 99) vs. 85 (73, 108) GGT: 55 (45, 91) vs. 77 (54, 123)	137 (96, 190) vs. 132 (100, 157)
			Cilofexor 30 mg + Firsocostat 20 mg, oral, once daily	Cilofexor 30 mg, oral, once daily	78	69	40	34	59.6 ± 9.2	65.3	94.2 ± 25.3/34.0 ± 7.2	71.2		AST:46 (29, 56) vs. 49 (35, 62) ALT: 42 (28, 65) vs. 50 (37, 66)	ALP: 86 (64, 102) vs. 95 (76, 127) GGT: 55 (45, 91) vs. 94 (54, 170)	147 (117,167) vs. 136 (97, 173)
Phase 2 Loomba et al. (2018) [23]	USA	Adults with NAFLD	Firsocostat 20 mg, oral, once daily	Placebo	49	46	26	26	55.4 ± 12.0	69.3	94.2 ± 21.0/ 31.9 ± 5.5	62.7	F1–F3 MRE ≥ 2.5 kPA			

BMI, body mass index; ELF, enhanced liver fibrosis; F, fibrosis; MRE, magnetic resonance elastography; NAFLD, nonalcoholic fatty liver disease; N/A, not available; SC, subcutaneous injection; T2DM, type 2 diabetes mellitus; TG, triglyceride; USA, United States of America; VCTE, vibration-controlled transient elastography; Continuous data presented as mean ± SD. ^a^ overweight/obese and having ≥2 of 5 NAFLD/NASH risk factors (fasting plasma glucose ≥ 100 mg/dL; fasting serum HDL-C  <  40 for males mg/dL or < 50 mg/dL for females; fasting serum triglycerides ≥ 150 mg/dL; blood pressure ≥ 130/85 mmHg; waist circumference ≥ 102 cm for males or ≥89 cm for females) or use of medications for these conditions.

**Table 2:** Outcomes of the included studies.

Author (Year)	Intervention (Regimen, Dose)	Control (Regimen, Dose)	Duration (Weeks)	Glucose Metabolism	Liver Biochemistry	Liver Fat MRI-PDFF Change (%)	Fibrosis and Other Markers of Liver Injury
						Proportion of ≥ 30% Reduction (%)	
Al Khouri et al. (2022) [24]	Semaglutide 0.24–24 mg (escalated dose over 16 weeks), SC, once weekly + Firsocostat 20 mg, oral, once daily	Semaglutide 0.24–24 mg (escalated dose over 16 weeks), SC, once weekly	24	FPG, mg/dL: LSM (95% CI) −32 (−40, −23) vs. −31 (−40, −23), *p* > 0.05 fasting insulin, μIU/mL: LSM (95% CI) −8.2 (−13.9, −2.5) vs. −8.5 (−14.6, −2.5), *p* > 0.05 HbA1c, %: LSM (95% CI) −1.2 (−1.4, −1.0) vs. −1.0 (−1.2, −0.7), *p* > 0.05 HOMA-IR: LSM (95% CI) 3.8 (−5.5, −2.1) vs. −3.5 (−5.3, −1.7), *p* > 0.05	AST, U/L: LSM (95% CI) −26 (−35, −18) vs. −11 (−2, −20), ***p* < 0.05** ALT, U/L: LSM (95% CI) −37 (−28, −45) vs. −13 (−3, −24), ***p* < 0.05**	LSM (95% CI) −11.6 (−9.3, −13.9) vs. −8.6 (−6.3, −10.9) ^a^, ***p* < 0.0353** 93.3 vs. 80.0	MRE, kPA: LSM (95% CI) −0.20 (−0.47, 0.06) vs. −0.13 (−0.40, 0.14), *p* > 0.05 ELF: LSM (95% CI) −0.59 (−0.87, −0.30) vs. −0.56 (−0.86, −0.27), *p* > 0.05 FibroSure/FibroTest: LSM (95% CI) −0.01 (−0.05, 0.03) vs. 0 (−0.04, 0.04), *p* > 0.05 CK18M30, U/L: LSM (95% CI) −312 (−381, −243) vs. −179 (−252, −107), ***p* < 0.05**
Calle et al. (2021 a) [22]	Clesacostat 25 mg, oral, once daily	Placebo	16	HbA1c, %: LSM (80% CI) −0.2 (−0.27, −0.14) vs. 0.03 (−0.03, 0.10) ^a^ T2DM HbA1c, %: LSM (80% CI) −0.2 (−0.33, −0.08) vs. 0.05 (−0.07, 0.18) ^a^	AST, %: LSM (80% CI) −15.6 (−21.5, −9.3) vs. −6.6 (−12.9, 0.1) ^b^ ALT, %: LSM (80% CI) −31.3 (−36.6, −25.5) vs. −8.5 (−15.2, −1.2) ^b^ ALP, %: LSM (80% CI) 15.5 (11.9, 19.1) vs. −0.8 (−3.8, 2.2) ^b^ GGT, %: LSM (80% CI) 21.5 (13.0, 30.6) vs. −7.7 (−13.9, −1.2) ^b^ total bilirubin: LSM (80% CI) −16.9 (−23.4, −10.0) vs. −13.7 (−20.3, −6.5) ^b^	LSM (80% CI) −55.9 (−59.0, −52.4) vs. −7.2 (−13.9, 0.0) ^b^, ***p* < 0.001** 85.0 vs. 5.0	VCTE, %: LSM (80% CI) −14.6 (−20.8, −8.0) ^b^ (CK18M30, %: LSM (80% CI) −40 vs. −8 ^b^ CK18M65, %: LSM (80% CI) −32 vs. −6 ^b^
	Clesacostat 10 mg, oral, once daily			HbA1c, %: LSM (80% CI) −0.11 (−0.17, −0.05) vs. 0.03 (−0.03, 0.10) ^a^ T2DM HbA1c, %: LSM (80% CI) −0.15 (−0.27, −0.03) vs. 0.05 (−0.07, 0.18) ^a^	AST, %: LSM (80% CI) −16.8 (−22.2, −11.0) vs. −6.6 (−12.9, 0.1) ^b^ ALT, %: LSM (80% CI) −27.7 (−32.9, −22.2) vs. −8.5 (−15.2, −1.2) ^b^ ALP, %: LSM (80% CI) 6.9 (3.9, 10.1) vs. −0.8 (−3.8, 2.2) ^b^ GGT, %: LSM (80% CI) 4.9 (−1.9, 12.2) vs. −7.7 (−13.9, −1.2) ^b^ total bilirubin, %: LSM (80% CI) 2.7 (−4.7, 10.6) vs. −13.7 (−20.3, −6.5) ^b^	LSM (80% CI) −49.9 (−53.3, −46.2) vs. −7.2 (−13.9, 0.0) ^b^, ***p* < 0.001** 74.0 vs. 5.0	VCTE, %: LSM (80% CI) −14.4 (−20.2, −8.3) ^b^ CK18M30, %: LSM (80% CI) −37 vs. −8 ^b^ CK18M65, %: LSM (80% CI) −33 vs. −6 ^b^
Calle et al. (2021 b) [22]	Clesacostat 15 mg + Ervogastat 300 mg, oral, twice daily	Ervogastat 300 mg, oral, twice daily	6	N/A	AST, %: LSM (90% CI) −3.9 (−10.3, 3.0) vs. 2.2 (−4.7, 9.6) ^b^ ALT, %: LSM (90% CI) −7.9 (−13.8, −1.6) vs. −4.2 (−10.3, 2.4) ^b^ ALP, %: LSM (90% CI) −3.9 (−10.3, 3.0) vs. 2.2 (−4.7, 9.6) ^b^ GGT, %: LSM (90% CI) 10.2 (2.7, 18.2) vs. −6.3 (−12.7, 0.5) ^b^	LSM (90% CI) −40.1 (−46.6, −32.9) vs. −30.1 (−38.0, −21.3) ^b^ 60.0 vs. 45.0	N/A
	Clesacostat 15 mg, oral, twice daily	Placebo		N/A	AST, %: LSM (90% CI) −0.0 (−6.7, 7.2) vs. 5.1 (−4.4, 15.7) ^b^ ALT, %: LSM (90% CI) −12.4 (−18.2, −6.2) vs. −3.0 (−11.8, 6.6) ^b^ ALP, %: LSM (90% CI) 11.2 (7.6, 14.9) vs. 2.9 (−1.8, 7.9) ^b^ GGT, %: LSM (90% CI) 22.6 (14.1, 31.8) vs. 0.0 (−9.8, 10.9) ^b^	LSM (90% CI) −40.0 (−47.0, −32.1) vs. 8.1 (−8.6, 27.9) ^b^ ***p* < 0.0001** 80.0 vs. 0.0	N/A
	Clesacostat 15 mg + Ervogastat 300 mg, oral, twice daily	Placebo		N/A	AST, %: LSM (90% CI) −3.9 (−10.3, 3.0) vs. 5.1 (−4.4, 15.7) ^b^ ALT, %: LSM (90% CI) −7.9 (−13.8, −1.6) vs. −3.0 (−11.8, 6.6) ^b^ ALP, %: LSM (90% CI) −0.7 (−3.9, 2.5) vs. 2.9 (−1.8, 7.9) ^b^ GGT, %: LSM (90% CI) 10.2 (2.7, 18.2) vs. 0.0 (−9.8, 10.9) ^b^	LSM (90% CI) −40.1 (−46.6, −32.9) vs. 8.1 (-8.6, 27.9) ^b^ ***p* < 0.0001** 60.0 vs. 0.0	N/A
Dandan et al. (2023) Lawitz et al. (2023) [25,26]	Firsocostat 20 mg, oral, once daily	Selonsertib 18 mg, oral, once daily	12	N/A	N/A	N/A	N/A
	Firsocostat 20 mg, oral, once daily	Cilofexor 30 mg, oral, once daily					
	Selonsertib 18 mg + Firsocostat 20 mg, oral, once daily	Selonsertib 18 mg, oral, once daily					
	Cilofexor 30 mg + Firsocostat 20 mg, oral, once daily	Cilofexor 30 mg, oral, once daily					
Loomba et al. (2021) [21]	Firsocostat 20 mg, oral, once daily	Placebo	48	FSG, mg/dL: LSM (95% CI) −1 (−13, 11) vs. 8 (−3, 20), *p* = 0.27 fasting insulin, μIU/mL: LSM (95% CI) −1.3 (−9.77, 7.16) vs. 5.89 (−2.26, 14.04), *p* = 0.22 HbA1c, %: LSM (95% CI) 0.1 (−0.2, 0.3) vs. 0.1 (−0.2, 0.3), *p* = 0.92 HOMA-IR: LSM (95% CI) −0.81(−4.12, 2.50) vs. 2.01(−1.19, 5.20), *p* = 0.22	AST, U/L: LSM (95% CI) −12 (−19, −5) vs. −4 (−10, 3), *p* = 0.074 ALT, U/L: LSM (95% CI) −16 (−25, −7) vs. −7 (−15, 1), *p* = 0.12 ALP, U/L: LSM (95% CI) −3 (−10, 16) vs. 0 (−12, 13), *p* = 0.78 GGT, U/L: LSM (95% CI) −20 (−43, 4) vs. −17 (−40, 5), *p* = 0.88 total bilirubin, mg/dL: LSM (95% CI) 0.0 (0.0, 0.1) vs. 0.0 (0.0, 0.1), *p* = 0.80 total bile acids, μmol/L: LSM (95% CI) −0.9 (−3.7, 1.8) vs. 1.9 (−0.7, 4.5), *p* = 0.14	LSM (95% CI) −2.96 (−5.67, −0.24) vs. 0.96 (−1.46, 3.39) ^a^; ***p* = 0.033** N/A	MRE, kPA: LSM (95% CI) −0.79 (−1.84, 0.27) vs. 0.43 (−0.55, 1.40), *p* = 0.092 VCTE, kPA: LSM (95% CI) −6.3 (−9.6, −3.0) vs. −1.2 (−4.1, 1.8), ***p* = 0.021** ELF: LSM (95% CI) −0.1 (−0.4, 0.1) vs. 0.3 (0.1, 0.6), ***p* = 0.010** CK18 M30, U/L: LSM (95% CI) −105 (−203, −7) vs. −0 (−93, 93), *p* = 0.12 CK18 M65, U/L: LSM (95% CI) −149 (−351, 53) vs. −93 (−285, 100), *p* = 0.69
	Cilofexor 30 mg + Firsocostat 20 mg, oral, once daily	Placebo	48	FSG, mg/dL: LSM (95% CI) −1 (−7, 10) vs. 8 (−3, 20), *p* = 0.33 fasting insulin, μIU/mL: LSM (95% CI) −5.83 (−11.74, 0.08) vs. 5.89 (−2.26, 14.04), ***p* = 0.02** HbA1c, %: LSM (95% CI) 0.0 (−0.1, 0.2) vs. 0.1 (−0.2, 0.3), *p* = 0.77 HOMA-IR: LSM (95% CI) −1.21 (−3.57, 1.15) vs. 2.01 (−1.19, 5.20), *p* = 0.11	AST, U/L: LSM (95% CI) 12 (−17, −7) vs. −4 (−10, 3), ***p* = 0.050** ALT, U/L: LSM (95% CI) −18 (−24, −12) vs. −7 (−15, 1), ***p* = 0.033** ALP, U/L: LSM (95% CI) 19 (10, 29) vs. 0 (−12, 13), ***p* = 0.017** GGT, U/L: LSM (95% CI) −19 (−36, −2) vs. −17 (−40, 5), *p* = 0.91 total bilirubin, mg/dL: LSM (95% CI) −0.1 (−0.1, 0.0) vs. 0.0 (0.0, 0.1), ***p=* 0.010** total bile acids, μmol/L: LSM (95% CI) −2.7 (−4.6, −0.8) vs. 0.9 (−3.7, 1.8) vs. 1.9 (−0.7, 4.5), ***p* = 0.005**	LSM (95% CI) −4.00 (−6.01, −1.98) vs. 0.96 (−1.46, 3.39) ^a^; ***p* = 0.002** N/A	MRE, kPA: LSM (95% CI) 0.03 (−0.77, 0.82) vs. 0.43 (−0.55, 1.40), *p* = 0.52 VCTE, kPA: LSM (95% CI) −4.2 (−6.5, −1.9) vs. −1.2 (−4.1, 1.8), *p* = 0.10 ELF: LSM (95% CI) −0.0 (−0.2, 0.2) vs. 0.3 (0.1, 0.6), ***p* = 0.024** CK18 M30, U/L: LSM (95% CI) −158 (−226, −90) vs. −0 (−93, 93), ***p* = 0.006** CK18 M65, U/L: LSM (95% CI) −324 (−464, −184) vs. −93 (−285, 100), *p* = 0.053
	Cilofexor 30 mg + Firsocostat 20 mg, oral, once daily	Cilofexor 30 mg, oral, once daily	48	FSG, mg/dL: LSM (95% CI) −1 (−7, 10) vs. 2 (−9, 14) fasting insulin, μIU/mL: LSM (95% CI) −5.83 (−11.74, 0.08) vs. −0.50 (−8.62, 7.61) HbA1c, %: LSM (95% CI) 0.0 (−0.1, 0.2) vs. 0.1 (−0.2, 0.4) HOMA-IR: LSM (95% CI) −1.21 (−3.57, 1.15) vs. 1.06 (−2.12, 4.24)	AST, U/L: LSM (95% CI) 12 (−17, −7) vs. −4 (−11, 3) ALT, U/L: LSM (95% CI) −18 (−24, −12) vs. −12 (−21, −4) ALP, U/L: LSM (95% CI) 19 (10, 29) vs. 1 (−12, 14) GGT, U/L: LSM (95% CI) −19 (−36, −2) vs. −37 (−60, −14) total bilirubin, mg/dL: LSM (95% CI) −0.1 (−0.1, 0.0) vs. 0.0 (−0.1, 0.0) total bile acids, μmol/L: LSM (95% CI) −2.7 (−4.6, −0.8) vs. −0.0 (−2.7, 2.7)	LSM (95% CI) −4.00 (−6.01, −1.98) vs. −3.04 (−6.43, 0.36) ^a^ N/A	MRE, kPA: LSM (95% CI) 0.03 (−0.77, 0.82) vs. 0.08 (−1.37, 1.53) VCTE, kPA: LSM (95% CI) −4.2 (−6.5, −1.9) vs. −4.3 (−7.5, −1.0) ELF: LSM (95% CI) −0.0 (−0.2, 0.2) vs. 0.2 (−0.1, 0.4) CK18 M30, U/L: LSM (95% CI) −158 (−226, −90) vs. 26 (−71, 124) CK18 M65, U/L: LSM (95% CI) −324 (−464, −184) vs. −23 (−226, 180)
Loomba et al. (2018) [23]	Firsocostat 20 mg, oral, once daily	Placebo	12	Glucose, mg/dL: LSM (95% CI) 3.3 (−13.8, 20.4), *p* = 0.70 ^c^ Insulin, μIU/mL: LSM (95% CI) 2.79 (−16.6, 22.2), *p* = 0.78 ^c^ HbA1c, %: LSM (95% CI) 0.11 (−0.23, 0.44), *p* = 0.53 ^c^	AST, %: Median −5 vs. −3 ^b^, *p* = 0.60 ALT, %: Median −20 vs. −7 ^b^, *p* = 0.18 ALP, %: Median 9 vs. −5 ^b^, ***p* < 0.001** GGT, %: Median −4 vs. −8 ^b^, *p* = 0.13	Median (IQR) −29 (−48, −12) vs. −8 (−18, 10) ^b^, ***p* = 0.002** 47.8 vs. 15.4 ***p* = 0.004**	MRE, %: Median (IQR) −6 (−17, 8) vs. −13 (−23, −2) ^b^, *p* = 0.10 TE, %: Median (IQR) −7.2 (−32.6, 7.8) vs. 30.6 (4.5, 63.8) ^b^, ***p* = 0.032** TIMP-1, %: Median −7 vs. 1 ^b^, ***p* = 0.022** P-III-NP, %: Median −13 vs. −0.3 ^b^, ***p* = 0.011** HA, %: Median −6 vs. −15 ^b^ *p* = 0.39

ALP, alkaline phosphatase; ALT, alanine aminotransferase; AST, aspartate aminotransferase; CK18M30, cytokeratin 18 M30; ELF, enhanced liver fibrosis; GGT, gamma-glutamyl transferase; FPG, fasting plasma glucose; FSG, fasting serum glucose; HA, hyaluronic acid; HbA1c, Hemoglobin A1c; HOMA-IR, homeostatic model assessment of insulin resistance; IQR, interquartile range; LSM, least square means; MRE, Magnetic resonance elastography; MRI-PDFF, magnetic resonance imaging proton density fat fraction; N/A, not available; P-III-NP, Procollagen III-N-terminal peptide; TE, transient elastography; TIMP-1, Tissue Inhibitor of Metalloproteinases-1; VCTE, vibration-controlled transient elastography. ^a^ absolute change; ^b^ percentage of relative change; ^c^ *p*-value for comparison of LSM between firsocostat with placebo. Bold for *p* < 0.05.

### 3.3. Quality Assessment of Included Studies

**Figure 2:**
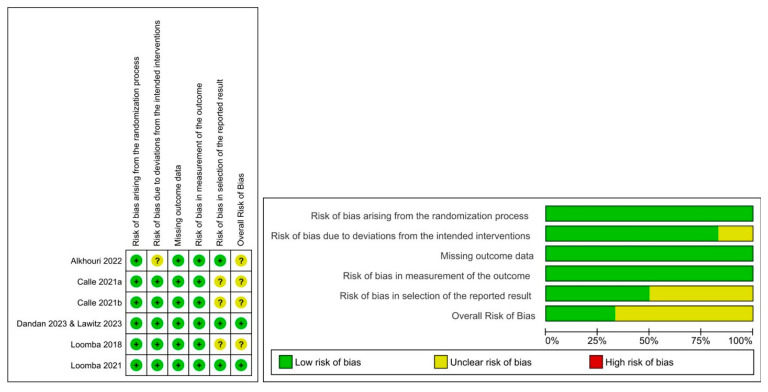
Quality assessment results of the ROB-2 tool; risk of bias graph [21,22,23,24,25,26].

The overall risk of bias for each study was assessed using the Cochrane ROB-2 tool, as illustrated in Figure 2. All studies were judged to have a low risk of bias across three domains: randomization process, missing outcome data, and measurement of the outcome. They adequately described details of their randomization procedures, including appropriate allocation concealment, thereby minimizing selection bias. There were no major deviations from the intended interventions, and any missing data were appropriately addressed using the prespecified method. Each study adhered to a prespecified protocol for both the conduct and analysis phases. Although minor concerns were raised about the possibility of multiple eligible outcome analyses, these were mitigated by prespecified analysis plans and detailed justifications provided in trial registries and published protocols. The low risk of bias across all included studies strengthens the internal validity of the pooled effect estimates and reduces the likelihood of systematic errors. Consequently, the robustness of the synthesized results can be assured.

### 3.4. Outcome Differences Between ACC Inhibitors and Placebo Groups on Steatosis, Fibrosis, and Liver Biochemistry Markers

#### 3.4.1. MRI-PDFF and MRE

**Figure 3:**
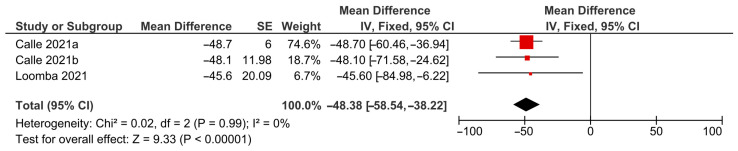
Forest plot of meta-analysis of the ACC inhibitors vs. placebo in NAFLD patients on MRI-PDFF [21,22]. The black diamond shows the overall pooled mean difference and its 95 % CI. A diamond not crossing the null line signifies a statistically significant overall effect (*p* < 0.05). CI: confidence interval; MD: mean difference between intervention and control groups on the scale of [units of percentage]; IV: Inverse-Variance; df: degrees of freedom; I^2^: percentage of total variation across studies (heterogeneity); SE: standard error.

Three studies were included in the meta-analysis. The forest plot (Figure 3) presents the pooled findings obtained using a fixed-effects model comparing the ACC inhibitor monotherapy versus placebo on MRI-PDFF. The percentage reduction in liver fat content was significantly higher in the ACC inhibitor groups than in the placebo groups (MD: −48.38; 95% CI: −58.54 to −38.22; *p* < 0.0001). No heterogeneity was observed among the included studies (I^2^ = 0%), indicating good consistency in treatment effect. The leave-one-out sensitivity analysis revealed that no single study affected the significance of the pooled effect size. Considering that a ≥30% relative reduction in MRI-PDFF is generally accepted as the threshold for meaningful steatosis improvement [27], the pooled estimate clearly exceeds this threshold, supporting both clinical and statistical significance.

MRE outcomes were not included in the quantitative synthesis due to the limited number of eligible studies and substantial methodological heterogeneity. Variations in MRE acquisition protocols, stiffness thresholds, reporting units, and baseline population characteristics precluded meaningful data aggregation and reliable comparative analysis. Loomba et al. (2018) evaluated firsocostat 20 mg for 12 weeks in patients with NAFLD and fibrosis stages F1–F3 (baseline median MRE: 3.40 kPA [IQR 2.96–3.99] vs. 3.46 kPA [3.15–4.20]) [23]. In contrast, Loomba et al. (2021) investigated the same agent over 48 weeks in patients with more advanced fibrosis (F3–F4; baseline median MRE: 5.8 kPA [4.4–6.6] vs. 5.0 kPA [4.2–7.0]) [21]. These differences in treatment duration and baseline disease stage may impact the magnitude of liver stiffness reduction. Loomba et al. (2021) showed a non-significant trend toward reduced stiffness with the ACC inhibitor (LSM change −0.79 kPA [95% CI −1.84 to 0.27] vs. 0.43 kPA [−0.55 to 1.40]; *p* = 0.092) [21], whereas Loomba et al. (2018) reported a numerically greater reduction in the placebo group (median −13% [IQR −23 to −2] vs. −6% [IQR −17 to 8]; *p* = 0.10) [23]. Neither study demonstrated statistically significant or clinically meaningful fibrosis regression.

#### 3.4.2. Liver Biochemistry

**Figure 4:**
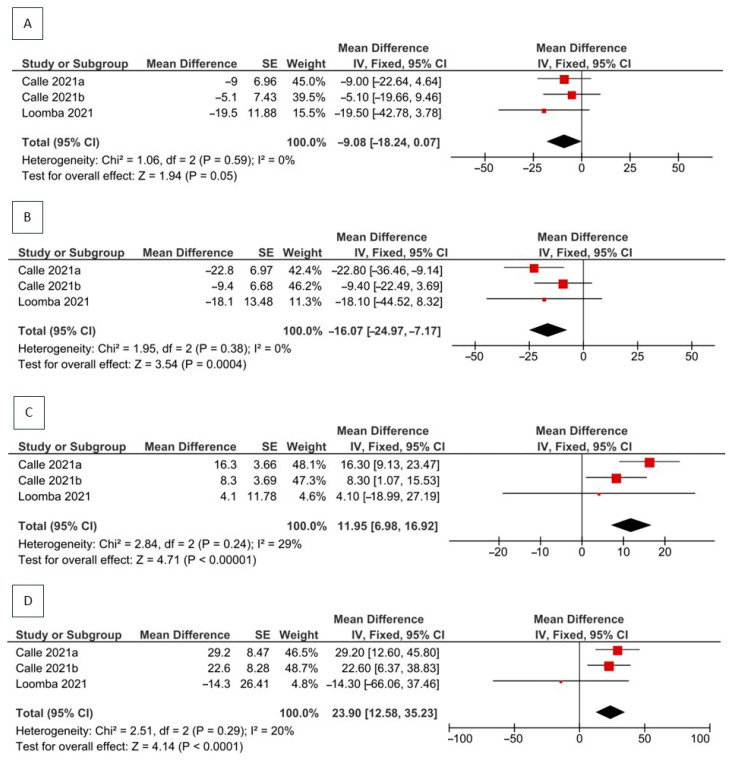
Forest plot of meta-analysis of the ACC inhibitors vs. placebo in NAFLD patients [21,22]; The black diamond shows the overall pooled mean difference and its 95 % CI. A diamond not crossing the null line signifies a statistically significant overall effect (*p* < 0.05). AST (**A**); ALT (**B**); ALP (**C**); GGT (**D**). CI: confidence interval; MD: mean difference between intervention and control groups on the scale of [units of percentage]; IV: Inverse-Variance; df: degrees of freedom; I^2^: percentage of total variation across studies (heterogeneity); SE: standard error.

Figure 4 demonstrates that ACC inhibitor monotherapy was associated with greater percentage reductions in AST and ALT compared with placebo (MD: −9.08%, 95% CI: −18.24 to −0.07; *p* = 0.05; MD: −16.07%, 95% CI: −24.97 to −7.17; *p* = 0.0004), although statistical significance was only observed for ALT. No heterogeneity was observed for either outcome (I^2^ = 0%), indicating good consistency among studies. Calle et al. (2021 a) showed greater changes in liver enzyme levels than Calle et al. (2021 b), probably due to longer treatment duration and higher cumulative dose in the former (clesacostat 25 mg/day for 16 weeks vs. 15 mg twice daily for 6 weeks) [22]. These findings may reflect the hepatoprotective effect of ACC inhibitors in mitigating liver injury associated with NAFLD progression. Supporting this, Alkhouri et al. (2022) reported significant reductions in AST and ALT levels alongside decreased cytokeratin-18 M30 (CK18M30) levels, a biomarker of apoptotic hepatocytes [24]. Similarly, Loomba et al. (2021) observed concurrent reductions in AST/ALT and CK18M30, accompanied by improvements in liver stiffness measurements [21].

Pooled estimates for ALP and GGT showed significantly higher levels in the ACC inhibitor groups compared with placebo (MD: 11.95%, 95% CI: 6.98 to 16.92; *p* < 0.00001, I^2^ = 29% and MD: 23.90%, 95% CI: 12.58 to 35.23; *p* < 0.0001, I^2^ = 27%, respectively), suggesting the effect of ACC inhibitors in elevating cholestatic liver enzymes. Such elevations may reflect an on-target pharmacological effect of ACC inhibition on biliary physiology or cholangiocyte activity rather than hepatocellular injury per se [28]. Although the magnitude of increase varied between ALP and GGT, the relatively low heterogeneity (I^2^ < 30%) suggests a consistent effect across studies. Interestingly, Loomba et al. (2021) reported smaller increases in both enzymes than other studies, probably due to differences in the ACC inhibitor used, the extended treatment duration, or the more advanced fibrosis stage in their studied population treated with firsocostat 20 mg for 48 weeks [21]. Leave-one-out sensitivity analysis showed that no individual study altered the statistical significance of the pooled AST, ALP, or GGT estimates. However, the pooled ALT result was sensitive to the exclusion of Calle et al. (2021a) [22]: its removal rendered the effect non-significant (*p* = 0.06), highlighting this study’s influence on the robustness of the finding.

### 3.5. Safety Outcomes

Treatment-emergent adverse events and laboratory abnormalities are summarized in Table 3. No mortality was reported in any ACC inhibitor-treated arm across all RCTs. Overall, the incidence of TEAEs was slightly higher in the firsocostat and clesacostat arms than in the placebo. Serious adverse events (SAEs) were infrequent but occurred more often in the ACC inhibitors group. These included two cases of cardiac disorders in the clesacostat 25 mg group [22], and two cases of acute myocardial infarction in the firsocostat 20 mg group and firsocostat 20 mg + Cilofexor 30 mg group [21]. The firsocostat 20 mg group showed a modest increase in non-serious TEAEs relative to placebo. Similarly, the clesocostat 25 mg group, but not the lower dose one, had a slightly higher incidence of non-serious TEAEs than placebo [21,22,23]. Semaglutide combined with firsocostat resulted in a slightly higher incidence of TEAEs than semaglutide alone. However, the combined regimen produced no incidence of SAE, while one case of SAE was reported in the semaglutide monotherapy group [24]. Combinations of clesacostat with Ervogastat, firsocostat with selonsertib, or Cilofexor showed comparable TEAE frequencies to their respective control group [22].

**Figure 5:**
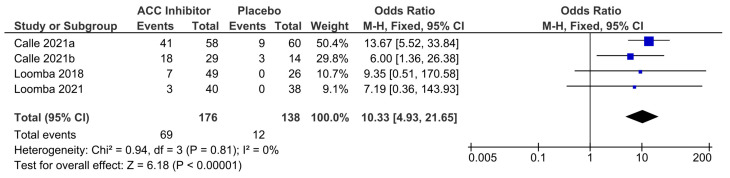
Forest plot summarizes the proportions of hypertriglyceridemia, comparing ACC inhibitor versus placebo in NAFLD patients [21,22,23]. It was pooled under a Mantel–Haenszel fixed-effects model. The black diamond shows the overall pooled proportion and its 95 % CI. A diamond not crossing the null line signifies a statistically significant overall effect (*p* < 0.05). CI = confidence interval; OR: odds ratio; MH = Mantel–Haenszel; df: degrees of freedom; I^2^: percentage of total variation across studies (heterogeneity).

Monotherapy with either firsocostat or clesacostat was associated with a higher incidence of TELAs [21,22]. In particular, the incidence of hypertriglyceridemia was significantly higher in the ACC inhibitor monotherapy group compared with placebo (OR = 10.33, 95% CI 4.93–21.65; *p* < 0.00001), indicating a more than tenfold increased risk associated with treatment. The forest plot (Figure 5) summarizes the proportions of hypertriglyceridemia events across studies, with no observed heterogeneity (I^2^ = 0%), suggesting a consistent finding. However, variations in agent, dosage, treatment duration, and the cut-off used to define hypertriglyceridemia may have influenced the OR estimate of the individual study. For instance, administration of clesacostat 25 mg daily for 16 weeks resulted in a twofold higher risk compared with clesacostat 15 mg twice daily for 6 weeks [22]. Loomba et al. (2018) also documented a significant increase in triglyceride levels from baseline in the firsocostat-treated group, relative to placebo (*p* = 0.008) [23]. The hypertriglyceridemic effect of ACC inhibitors appears dose-dependent and clinically relevant. Notably, co-administration with Ervogastat was shown to mitigate this effect [22].

## 4. Discussion

This meta-analysis has shown that the dual ACC 1/2 inhibitors, either given singly or combined with other classes of agents, significantly reduced liver fat content after at least 6 weeks of administration. This was shown by MRI-PDFF, a non-invasive test with high accuracy and reliability that can be an alternative to liver biopsy for evaluating hepatic steatosis in patients with NAFLD, either in real clinical settings or trials [27]. One study revealed a considerable proportion of patients experiencing at least a 30% reduction in steatosis after 12 weeks of firsocostat 20 mg daily administration [23]. This finding is of clinical importance since a ≥30% decline in MRI-PDFF is associated with histologic response and a higher probability for NASH resolution and thus is employed as a cut-off to identify histologic responders in NASH trials [27]. In addition to the downregulation of DNL and increased fatty acid transfer to mitochondria for β-oxidation, a substantial elevation of Wnt5a protein is a proposed alternative mechanism for liver fat content reduction under ACC inhibition [29].

Studies by Loomba et al. (2018 and 2021) reported a significant improvement in fibrosis through a radiological assessment using TE and VCTE, respectively [21,23]. Loomba et al. (2021) also documented significant improvement in the ELF score—a composite biomarker incorporating TIMP-1, PIIINP, and HA levels [21]. Similarly, Loomba et al. (2018) observed significant reductions in individual biomarkers TIMP-1 and PIIINP [23]. Alkhouri et al. (2022) documented a significant decline in CK18M30 with firsocostat plus semaglutide administration compared with semaglutide alone [24]. Although these blood-based biomarkers are promising non-invasive tests for detecting fibrogenesis, their limited accuracy when used in isolation remains problematic [30]. None of the studies demonstrated a significant improvement in fibrosis as measured by MRE following firsocostat therapy, suggesting no clear benefit for fibrosis regression. The fibrosis stage is the most important indicator of disease severity and progression, guiding clinical decisions for patients with NAFLD [31]. Consequently, fibrosis regression has been designated a surrogate endpoint for therapeutic proof in the NASH trial [32]. Both MRE and VCTE are acceptable non-invasive methods to evaluate liver fibrosis in NAFLD; however, MRE is considered more accurate and reliable, particularly for assessing longitudinal treatment effect [32,33].

The dissociation of ACC inhibitors’ effect against steatosis from their effect on fibrosis in this meta-analysis can be explained by the notion that these are separate histological features with overlapping but distinct pathophysiological pathways. While fat can induce inflammation and contribute to fibrosis, once fibrosis is established, it may be sustained by ongoing inflammation, oxidative stress, and other cell-damaging processes, such as mitochondrial dysfunction, that persist even after hepatic fat content is reduced [34]. Ongoing mitochondrial dysfunction generates reactive oxygen species (ROS), which can sustain activation of hepatic stellate cells, promoting fibrogenesis [35]. Furthermore, steatosis is a reversible condition that improves rapidly under certain interventions, whereas fibrosis, especially at the advanced stage, tends to be irreversible [34]. Therefore, a more extended treatment period is likely required to achieve discernible improvement in fibrosis. In general, fibrosis regression can be attained after a minimum of one year of follow-up [32]. Nonetheless, firsocostat, alone or combined with Cilofexor, is still promising to be efficacious, at least in decelerating fibrosis progression in patients on the NAFLD spectrum, as was shown in a meta-analysis that concluded this agent was effective in improving liver stiffness measurement on VCTE and ELF [36]. Moreover, preclinical in vitro and in vivo assays have established dual ACC 1/2 inhibitors’ anti-fibrotic activity through mechanisms related or unrelated to intrahepatic DNL inhibition [9]. Inhibition of ACC1 and ACC2 enzymes directly blocks metabolic pathways accountable for hepatic stellate cell activation and proinflammatory T cell proliferation [37].

Findings of the secondary outcomes revealed conflicting effects of ACC inhibitors on liver enzymes. While reductions in AST and ALT levels were observed, only ALT reached statistical significance. Conversely, ALP and GGT were significantly elevated in the treatment arms compared with the placebo. Although ALT levels and ALT/AST ratio are useful indicators for predicting NAFLD and liver fibrosis, and are often assessed as treatment outcomes, changes in these parameters have a limited ability to reflect NASH resolution or fibrosis regression [38,39]. Therefore, reductions in AST and ALT should be interpreted with caution, since they do not necessarily reflect histological improvement, as suggested by inconsistent findings of fibrosis regression, despite a decline in transaminase levels in this study.

ALP and GGT are less frequently used for evaluating and monitoring therapy and have little clinical meaning in NAFLD diagnosis [38]. An increase in ALP and GGT was observed in clesacostat and firsocostat monotherapy studies. Of note, this statistical significance does not always imply clinical relevance. The mechanism of elevated ALP and GGT in this regard remains unclear [22]. The concurrent increase of ALP and GGT related to ACC inhibitor administration was possibly a benign, transient condition that would resolve with the cessation of therapy [39]. However, a possibility for the implication of an adverse effect of a cholestatic condition cannot be ruled out. Elevated cholestatic markers could signal potential alterations in bile acid metabolism or subclinical cholestasis during therapy that warrant important considerations for liver monitoring and safety in future studies [28].

In general, ACC inhibitors had no effects on the glucose metabolism profile. Only a study by Loomba et al. (2021) reported a significant reduction in fasting insulin levels in the firsocostat + Cilofexor-treated group [21]. This implies the potential utility of ACC inhibitors for NAFLD patients with type 2 DM. This meta-analysis confirmed the hypertriglyceridemic effect of ACC inhibitors, as was shown by the pooled OR estimate. All included studies consistently observed a clinically relevant increase in triglyceride levels. Dose-dependent hypertriglyceridemia was documented in an RCT evaluating clesocostat by Calle et al. (2021). This adverse effect is mitigated by concomitant administration of clesacostat with Ervogastat [22]. Firsocostat, either given alone or in combination with semaglutide, selonsertib, and Cilofexor, starting from a 12-week duration of administration and at a 5 mg dose, produced hypertriglyceridemia in a substantial proportion of subjects [21,23,24,25]. Other findings of lipid abnormality include significantly elevated VLDL and lowered HDL cholesterol [24]. Malonyl CoA depletion due to hepatic ACC inhibition results in the reduction of PUFA production, leading to SREBP-1c transactivation that upregulates glycerol-3-phosphate acyltransferase (GPAT)1 expression; consequently, hepatic triglyceride synthesis increases [40]. Additionally, SREBP-1c-mediated elevation of apolipoprotein C III also contributes to hypertriglyceridemia through inhibition of lipoprotein lipase, making the degradation of circulatory triglyceride-rich lipoproteins (TrLs) slow down, thus preventing their hepatic clearance [29,41]. By far, lipid abnormalities are the most critical issue negatively impacting the safety profile of ACC inhibitors for NAFLD therapy. Although mostly reported as asymptomatic in RCTs, emerging or worsening hypertriglyceridemia related to ACC inhibitors may aggravate cardiometabolic factors and increase cardiovascular risks in NAFLD [42]. The urgency for a nomenclature transition to MASLD nowadays emanates from the essential fact that this is a systemic metabolic disorder and that mortality cases due to CVD complications outnumber liver-related complications. Therefore, any therapeutic approach should clearly impose a beneficial or at least neutral effect on the CVD risk profile [43]. Combination therapy with Ervogastat or fenofibrate appears to mitigate ACC inhibitor-induced hypertriglyceridemia and is claimed to bring no adverse consequences [22,26]. The development of selective ACC inhibitors that, expectedly, safeguard cardiometabolic factors is underway and has not yet entered the clinical phase [42].

In comparison with other DNL-inhibiting agents subject to clinical investigations in NAFLD, ACC inhibitors apparently show superior efficacy in reducing liver fat content and ALT. A fatty acid synthase inhibitor, orlistat, produced a statistically significant but not clinically relevant reduction of liver fat content and AST/ALT levels [44,45]. Aramchol, an inhibitor of stearoyl CoA desaturase 1 enzyme, failed to show significant improvement in liver fat content. However, it did not produce hypertriglyceridemia [46]. Orlistat was similar to ACC inhibitors in terms of association with increased serum triglyceride levels [44].

To our knowledge, this is the first systematic review and meta-analysis of randomized controlled trials (RCTs) that clearly captures the efficacy of dual ACC 1/2 inhibitors in reducing liver fat, as measured by MRI-PDFF. The analysis also demonstrated a significant reduction in ALT levels following ACC inhibitor therapy. We rigorously evaluated both the statistical and clinical significance of these findings. Administration of firsocostat and clesacostat for at least 6 weeks resulted in statistically significant and clinically relevant hypertriglyceridemia, confirming pre-clinical studies and underscoring the safety concerns of ACC inhibitors in NAFLD patients.

The findings of this study suggest the potential utility of dual ACC 1/2 inhibitors in early NASH, a liver inflammatory condition with predominant steatosis and minimal fibrosis features [47], provided that the hypertriglyceridemia effect is mitigated. These agents should be exclusively given to normotriglyceridemic NAFLD patients. In the context of MASLD, monotherapy with an ACC inhibitor alone may not constitute a viable therapeutic option. This agent is promising to be an adjunct with a focus on hepatic steatosis reduction for MASH therapy. It is likely to be combined with antifibrotic agents working on distinct pathways, like resmetirom, a thyroid hormone receptor beta agonist, the only MASLD-specific agent that has received FDA approval [48], or one that has received breakthrough therapy designation, efruxifermin, a fibroblast growth factor 21 (FGF21) analogue [49].

A combinatorial approach is the emerging paradigm in MASLD therapy, capitalizing on synergistic effects across multiple pathways, as has been conceptualized in multi-hit theory [50]. Evidence from RCTs analyzed in this study, evaluating the addition of ACC 1/2 inhibitors to semaglutide, Ervogastat, or Cilofexor, demonstrates superior efficacy, at least in liver fat reduction, to monotherapy of the latter three agents, without compromised safety, indicating the future prospect of ACC inhibitor-based combinatory regimens [22,24,26]. Further RCTs of larger sample sizes and longer follow-ups are warranted to substantiate this claim. The combination of ACC inhibitors, either with fenofibrate or Ervogastat, was shown to effectively prevent hypertriglyceridemia, showing another benefit of a combinatorial approach in optimizing safety outcomes [22,25]. No clinically meaningful drug interaction-related adverse effects were reported in clinical trials evaluating ACC inhibitors combined with other classes of agents [21,24,51]. Of note, careful assessment of drug–drug interaction should be underscored in future studies evaluating ACC inhibitors in combinatory regimens, providing the transition from NAFLD to MASLD requires a different framework in managing the liver pathology within the broader landscape of systemic cardiometabolic diseases, which suggests polypharmacy becomes inevitable [52].

Several limitations of this study must be acknowledged. First, only a few studies adequately reported fibrosis using MRE. The relatively short follow-up periods may have limited the ability to detect improvements in fibrosis, suggesting a longer follow-up period is required in future studies. Second, we could not perform subgroup analyses based on specific drugs, dosages, or treatment durations, nor could we perform a funnel plot test for assessment of publication bias due to the limited number of available studies.

## 5. Conclusions

The administration of dual ACC 1/2 inhibitors, firsocostat and clesacostat, over 6 to 24 weeks leads to a reduction in liver fat content without any impact on fibrosis. These agents are associated with clinically relevant hypertriglyceridemia, raising concern about elevated cardiometabolic risk. As NAFLD transitions to the MASLD paradigm, emphasizing the critical roles of glucose and lipid metabolic dysfunction in liver disease progression, single administration of dual ACC inhibitors may not be the ideal therapeutic option, especially for patients with co-existing hypertriglyceridemia. Alternative strategies that safely and effectively inhibit de novo lipogenesis (DNL) while improving metabolic dysfunction and liver pathology are needed. Combinatorial therapy that includes ACC inhibitors and other agents that tackle the mechanistic axes of the multi-hit model should be explored in further robust studies.

## Figures and Tables

**Table 3:** Treatment-emergent adverse events (TEAEs) and treatment-emergent laboratory abnormalities (TELAs).

Author (Year)	Intervention	Control	Duration (Weeks)	TEAEs				TELAs
				All-Cause Mortality *n* (%)	SAE *n* (%)	Others *n* (%)	Total *n* (%)	
Al Khouri et al. (2022) [24]	Semaglutide 0.24–24 mg + Firsocostat 20 mg	Semaglutide 0.24–24 mg	24	-	0 (0.0) vs. 1 (4.8)	17 (77.3) vs. 16 (76.2)	22 (86.4) vs. 21 (81.0)	Grade ≥ 3: 1 (4.5%) vs. 0 (0%), hypertriglyceridemia (575 mg/dL) triglyceride change, mg/dL: LSM (95% CI), 15 (−16, 46) vs. −28 (−60, 4) HDL change, mg/dL: LSM (95% CI) −5 (−8, −3) vs. −1 (−3, 2), ***p* < 0.05** VLDL change, mg/dL: LSM (95% CI) 4 (0, 8) vs. −7 (−12, −2), ***p* < 0.05**
Calle et al. (2021 a) [22]	Clesacostat 25 mg	Placebo	16	-	2 (3.4) vs. 0 (0.0) Cardiac disorders	31 (53.4) vs. 27 (44.3)	45 (78) vs. 41 (67)	total: 42 (72%) vs. 11 (18%) hypertriglyceridemia (≥400 mg/dL): 41 (71%) vs. (9 (15%) triglyceride change, %: LSM (80% CI), 86.8 (72.9, 101.9) vs. 4.8 (−3.0, 13.3) ^b^
	Clesacostat 10 mg			-	1 (1.6) vs. 0 (0.0) Upper respiratory infection Injury	25 (40.3) vs. 27 (44.3)	42 (68) vs. 41 (67)	total: 40 (64%) vs. 11 (18%) hypertriglyceridemia (≥400 mg/dL): 38 (61%) vs. (9 (15%) triglyceride change, %: LSM (80% CI), 59.2 (48.0, 71.2) vs. 4.8 (−3.0, 13.3) ^b^
Calle et al. (2021 b) [22]	Clesacostat 15mg + Ervogastat 300mg	Ervogastat 300mg	6	-	1 (3.6) vs. 0 (0.0) jaw abscess	1 (3.6) vs. 6 (21.4)	10 (36) vs. 10 (36)	total: 3 (11%) vs. 4 (14%) hypertriglyceridemia (≥400 mg/dL): 3 (11%) vs. 3 (11%) triglyceride change, %: LSM (90% CI), 13.8 (2.3, 26.7) vs. −1.89 (−11.9, 9.3) ^b^
	Clesacostat 15mg	Placebo		-	-	6 (20.7) vs. 3 (21.4)	10 (35) vs. 3 (21)	total: 17 (59%) vs. 3 (43%) hypertriglyceridemia (≥400 mg/dL): 18 (62%) vs. 3 (21%) triglyceride change, %: LSM (90% CI), 58.2 (41.8, 76.5) vs. 7.4 (−8.1, 25.4) ^b^
	Clesacostat 15mg + Ervogastat 300mg	Placebo		-	1 (3.6) vs. 0 (0.0) jaw abscess	1 (3.6) vs. 3 (21.4)	10 (36) vs. 3 (21)	total: 3 (11%) vs. 3 (21%) hypertriglyceridemia (≥400 mg/dL): 3 (11%) vs. 3 (21%) triglyceride change, %: LSM (90% CI), 13.8 (2.3, 26.7) vs. 7.4 (−8.1, 25.4) ^b^
Dandan et al. (2023) Lawitz et al. (2023) [25,26]	Firsocostat 20 mg	Selonsertib 18 mg	12	-	-	6 (60) vs. 5 (50)	6 (60) vs. 5 (50)	grade ≥3: 2 (20%) vs. 2 (20%) hypertriglyceridemia: 1 (10%) vs. 0 (0%)
	Firsocostat 20 mg	Cilofexor 30 mg		-	-	6 (60) vs. 5 (50)	6 (60) vs. 5 (50)	grade ≥ 3: 2 (20%) vs. 4 (40%) hypertriglyceridemia: 1 (10%) vs. 0 (0%)
	Selonsertib 18 mg + Firsocostat 20 mg	Selonsertib 18 mg		-	1 (5) vs. 0 (0) Tooth abscess	8 (40) vs. 5 (50)	9 (45) vs. 5 (50)	grade ≥ 3: 4 (20%) vs. 2 (20%) hypertriglyceridemia: 1 (5%) vs. 0 (0%)
	Cilofexor 30 mg + Firsocostat 20 mg	Cilofexor 30 mg		-	1 (5) vs. 0 (0) UTI	10 (50) vs. 5 (50)	11 (55) vs. 5 (50)	grade ≥ 3: 2 (10%) vs. 4 (40%) no hypertriglyceridemia
Loomba et al. (2021) [21]	Firsocostat 20 mg	Placebo	48	-	3 (7.5) vs. 2 (5) ITP; AMI Pyrexia	30 (75) vs. 29 (74)	34 (85) vs. 31 (80)	total: 40 (100%) vs. 37 (95%) hypertriglyceridemia (>500 mg/dL): 3 (8%) vs. 0 (0%)
	Cilofexor 30 mg + Firsocostat 20 mg	Cilofexor 30 mg		-	8 (10) vs. 8 (20) AMI; Gastritis; Postprocedural hemorrhage; Hypoglycaemia; Diffuse large B-cell lymphoma; Cerebrovascular disorder; Urinary tract obstruction	66 (85) vs. 34 (85)	71 (91) vs. 37 (93)	total: 77 (100%) vs. 39 (97.5%) hypertriglyceridemia (>500 mg/dL): 3 (4%) vs. 0 (0%)
Loomba et al. (2018) [23]	Firsocostat 20 mg	Placebo	12	-	2 (4) vs. 0(0) Abdominal pain; Sepsis; Hepatic encephalopathy; Transient ischaemic attack;	29 (59) vs. 11 (42)	35 (71) vs. 16 (61)	hypertriglyceridemia (>500 mg/dL): 7 (14%) vs. 0 (0%) triglyceride change, mg/dL: LSM (95% CI), 97.2 (26.48, 168.97) ^a^, ***p* = 0.008** ^c^

AMI, acute myocardial infarction; HDL, high-density lipoprotein; ITP, idiopathic thrombocytopenic purpura; LSM, least square means; SAE, serious adverse event; TEAEs, treatment-emergent adverse events; TELAs, treatment-emergent laboratory abnormalities; UTI, urinary tract infection; VLDL, very low-density lipoprotein. ^a^ absolute change; ^b^ percentage of relative change; ^c^ *p*-value for comparison of LSM between firsocostat with placebo. Bold for *p* < 0.05.

## Supplementary Materials

The following supporting information can be downloaded at https://www.mdpi.com/article/10.3390/ph18091276/s1, Table S1. Clinical questions according to PICO; Table S2. Keywords of database searching.

## Abbreviations

The following abbreviations are used in this manuscript:

ACC	Acetyl-CoA carboxylase
ALP	Alkaline phosphatase
ALT	Alanine aminotransferase
AST	Aspartate aminotransferase
CK18M30	Cytokeratin 18 M30
DNL	De novo lipogenesis
ELF	Enhanced liver fibrosis
GGT	Gamma-glutamyl transferase
MASLD	Metabolic dysfunction-associated steatotic liver disease
MRE	Magnetic resonance elastography
MRI-PDFF	Magnetic resonance imaging proton density fat fraction
NAFLD	Nonalcoholic fatty liver disease
RCT	Randomized controlled trial
SAE	Serious adverse events
TE	Transient elastography
TEAE	Treatment-emergent adverse event
TELA	Treatment-emergent laboratory abnormalities
TIMP-1	Tissue Inhibitor of Metalloproteinases-1
VCTE	Vibration-controlled transient elastography
