# Supplementary material for: Acetyl-CoA Carboxylase Inhibitors for Nonalcoholic Fatty Liver Disease: A Systematic Review and Meta-Analysis of Randomized Controlled Trials
Source: Pharmaceuticals (Basel). 2025 Aug 27;18(9):1276. doi: 10.3390/ph18091276 (PMC12472739; doi:10.3390/ph18091276)
Supplement: Supplementary file 1 [file pharmaceuticals-18-01276-s001.zip › pharmaceuticals-3704220-supplementary.pdf]

Supplementary Table S1. Clinical questions according to PICO

Aspects	Criteria
Population (P)	-Adult patients with NAFLD diagnosis, established by liver biopsy or radiological examination, either accompanied by evident metabolic dysfunctions or not.<br>-No present hepatitis caused by viral infection, significant alcohol consumption, autoimmune liver disease, drug-induced liver injury, Wilson's disease, hemochromatosis, and severe malnutrition.<br>-Adult patients with MASLD diagnosis, established by liver biopsy or radiological examination, accompanied by at least one of five cardiometabolic risk(s).<br>-No transplantation history<br>-No malignant tumors<br>-Not in pregnancy or lactating period
Intervention (I)	Acetyl CoA inhibitors administered as a single agent or in combination with other classes of agents
Control (C)	Other agents, placebo
Outcome (O)	Primary outcomes: Changes in liver fat content and fibrosis measured by MRI-PDFF and MRE<br>Secondary outcomes:<br>-Other measures of liver fibrosis: VCTE, ELF score, fibrotest score, TIMP-1<br>-Liver enzymes: AST, ALT, ALP, GGT<br>-Bilirubin<br>-Blood glucose, HbA1c, Insulin, lipid panel<br>-Treatment-emergent adverse events<br>-Treatment-emergent laboratory abnormalities

Supplementary Table S2. Keywords of database searching

Database	Keywords	
PubMed	#1	"Non-alcoholic Fatty Liver Disease"[Mesh] OR "Nonalcoholic fatty liver disease*"[All Fields] OR "Non-alcoholic fatty liver disease*"[All Fields] OR "NAFLD"[All Fields] OR "Nonalcoholic steatohepatitis"[All Fields] OR "Non-alcoholic steatohepatitis"[All Fields] OR "NASH"[All Fields] OR "Metabolic dysfunction-associated steatotic liver disease"[Mesh] OR "Metabolic dysfunction-associated steatotic liver disease*"[All Fields] OR "MASLD"[All Fields] OR "Metabolic dysfunction-associated fatty liver disease*"[All Fields] OR "MAFLD"[All Fields] OR "Metabolic dysfunction-associated steatohepatitis"[All Fields] OR "MASH"[All Fields]
	#2	"Acetyl-CoA Carboxylase"[Mesh] OR "Acetyl-CoA carboxylase"[All Fields] OR "ACC inhibitor"[All Fields] OR "GS-0976"[All Fields] OR "NDI-010976"[All Fields] OR "ND-630"[All Fields] OR "PF-05221304"[All Fields] OR "Firsocostat"[All Fields]

	#3	"Clinical Trial" [Publication Type] OR "Controlled Clinical Trial" [Publication Type] OR "Random Allocation"[Mesh] OR "RCT"[All Fields] OR "random*"[All Fields] OR "trial*"[All Fields]
	#4	#1 AND #2 AND #3
Scopus	#1	TITLE-ABS-KEY("Nonalcoholic fatty liver disease*" OR "Non-alcoholic fatty liver disease*" OR "NAFLD" OR "Nonalcoholic steatohepatitis" OR "Non-alcoholic steatohepatitis" OR "NASH" OR "Metabolic dysfunction-associated steatotic liver disease" OR "Metabolic dysfunction-associated steatotic liver disease*" OR "MASLD" OR "Metabolic dysfunction-associated fatty liver disease*" OR "MAFLD" OR "Metabolic dysfunction-associated steatohepatitis" OR "MASH")
	#2	TITLE-ABS-KEY("Acetyl-CoA carboxylase" OR "ACC inhibitor" OR "GS-0976" OR "NDI-010976" OR "ND-630" OR "PF-05221304" OR "Firsocostat")
	#3	TITLE-ABS-KEY("RCT" OR "random*" OR "trial*")
	#4	#1 AND #2 AND #3
Web of Science	#1	ALL=("Nonalcoholic fatty liver disease*" OR "Non-alcoholic fatty liver disease*" OR "NAFLD" OR "Nonalcoholic steatohepatitis" OR "Non-alcoholic steatohepatitis" OR "NASH" OR "Metabolic dysfunction-associated steatotic liver disease" OR "Metabolic dysfunction-associated steatotic liver disease*" OR "MASLD" OR "Metabolic dysfunction-associated fatty liver disease*" OR "MAFLD" OR "Metabolic dysfunction-associated steatohepatitis" OR "MASH")
	#2	ALL=("Acetyl-CoA carboxylase" OR "ACC inhibitor" OR "GS-0976" OR "NDI-010976" OR "ND-630" OR "PF-05221304" OR "Firsocostat")
	#3	ALL=("RCT" OR "random*" OR "trial*")
	#4	#1 AND #2 AND #3
ProQuest	#1	mesh.Exact("Non-alcoholic Fatty Liver Disease")
	#2	noft("Nonalcoholic fatty liver disease*" OR "Non-alcoholic fatty liver disease*" OR "NAFLD" OR "Nonalcoholic steatohepatitis" OR "Non-alcoholic steatohepatitis" OR "NASH")
	#3	mesh.Exact("Metabolic dysfunction-associated steatotic liver disease")
	#4	noft("Metabolic dysfunction-associated steatotic liver disease" OR "Metabolic dysfunction-associated steatotic liver disease*" OR "MASLD" OR "Metabolic dysfunction-associated fatty liver disease*" OR "MAFLD" OR "Metabolic dysfunction-associated steatohepatitis" OR "MASH")
	#5	mesh.Exact("Acetyl-CoA Carboxylase")
	#6	noft("Acetyl-CoA carboxylase" OR "ACC inhibitor" OR "GS-0976" OR "NDI-010976" OR "ND-630" OR "PF-05221304" OR "Firsocostat")
	#7	mesh.Exact("Controlled Clinical Trial as Topic" OR "Randomized Controlled Trial as Topic" OR "Random Allocation")
	#8	noft("RCT" OR "random*" OR "trial*")
	#9	(#1 OR #2 OR #3 OR #4) AND (#5 OR #6) AND (#7 OR #8)
CINAHL (EBSCOhost)	#1	TX ("Nonalcoholic fatty liver disease*" OR "Non-alcoholic fatty liver disease*" OR "NAFLD" OR "Nonalcoholic steatohepatitis" OR "Non-alcoholic steatohepatitis" OR "NASH" OR "Metabolic dysfunction-associated steatotic liver disease" OR "Metabolic

		dysfunction-associated steatotic liver disease*” OR “MASLD” OR “Metabolic dysfunction-associated fatty liver disease*” OR “MAFLD” OR “Metabolic dysfunction-associated steatohepatitis” OR “MASH”)
	#2	TX ("Acetyl-CoA carboxylase" OR "ACC inhibitor" OR "GS-0976" OR "NDI-010976" OR “ND-630” OR “PF-05221304” OR “Firsocostat”)
	#3	TX ("RCT" OR "random*" OR "trial*")
	#4	#1 AND #2 AND #3
CENTRAL	#1	MeSH descriptor: [Non-alcoholic fatty liver disease] explode all trees
	#2	MeSH descriptor: [Metabolic dysfunction-associated steatotic liver disease] explode all trees
	#3	“Nonalcoholic fatty liver disease*” OR “Non-alcoholic fatty liver disease*” OR “NAFLD” OR “Nonalcoholic steatohepatitis” OR “Non-alcoholic steatohepatitis” OR “NASH” **OR “**Metabolic dysfunction-associated steatotic liver disease” OR “Metabolic dysfunction-associated steatotic liver disease*” OR “MASLD” OR “Metabolic dysfunction-associated fatty liver disease*” OR “MAFLD” OR “Metabolic dysfunction-associated steatohepatitis” OR “MASH”
	#4	MeSH descriptor: [Acetyl-CoA carboxylase] explode all trees
	#5	"Acetyl-CoA carboxylase" OR "ACC inhibitor" OR "GS-0976" OR "NDI-010976" OR “ND-630” OR “PF-05221304” OR “Firsocostat”
	#6	MeSH descriptor: [Random Allocation] explode all trees
	#7	MeSH descriptor: [Controlled Clinical Trials as Topic] explode all trees
	#8	MeSH descriptor: [Randomized Controlled Trials as Topic] explode all trees
	#9	"RCT" OR "randomized" OR "trial"
	#10	#1 OR #2 OR #3
	#11	#4 OR #5
	#12	#6 OR #7 OR #8 OR #9
	#13	#10 AND #11 AND #12
